# 1,1,2,2-Tetra­phenyl-1λ^5^-diphosphane 1-sulfide

**DOI:** 10.1107/S1600536809002955

**Published:** 2009-01-28

**Authors:** Bhaskar R. Aluri, Stephan Peitz, Anina Wöhl, Normen Peulecke, Bernd H. Müller, Anke Spannenberg, Uwe Rosenthal

**Affiliations:** a Leibniz-Institut für Katalyse e. V. an der Universität Rostock, Albert-Einstein-Strasse 29a, 18059 Rostock, Germany; b Linde AG, Linde Engineering Division, Dr.-Carl-von-Linde-Strasse 6–14, 82049 Pullach, Germany

## Abstract

In the title mol­ecule, C_24_H_20_P_2_S, the P—P bond length is 2.2263 (5) Å. The two phenyl rings attached to the three- and five-coordinated P atoms, respectively, form dihedral angles of 56.22 (5) and 71.74 (5)°.

## Related literature

For the literature on related compounds, see: Bhattacharyya *et al.* (1996 ▶); Gruber *et al.* (1990 ▶); Jones *et al.* (2002 ▶).
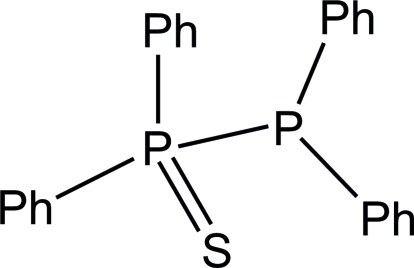


## Experimental

### 

#### Crystal data

C_24_H_20_P_2_S
*M* *_r_* = 402.40
Monoclinic, 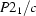

*a* = 9.32670 (19) Å
*b* = 13.6496 (4) Å
*c* = 16.0484 (4) Å
β = 91.7298 (17)°
*V* = 2042.12 (9) Å^3^
*Z* = 4
Mo *K*α radiation
μ = 0.32 mm^−1^
*T* = 200 (2) K
0.45 × 0.30 × 0.28 mm

#### Data collection

Stoe IPDS II diffractometer
Absorption correction: numerical (*X-SHAPE*; Stoe & Cie, 2005 ▶) *T*_min_ = 0.892, *T*_max_ = 0.961
38972 measured reflections
5499 independent reflections
4493 reflections with *I* > 2σ(*I*)
*R*_int_ = 0.027

#### Refinement

*R*[*F* ^2^ > 2σ(*F* ^2^)] = 0.033
*wR*(*F* ^2^) = 0.096
*S* = 1.08
5499 reflections
244 parameters
1 restraint
H-atom parameters constrained
Δρ_max_ = 0.29 e Å^−3^
Δρ_min_ = −0.28 e Å^−3^

### 

Data collection: *X-AREA* (Stoe & Cie, 2005 ▶); cell refinement: *X-AREA*; data reduction: *X-RED* (Stoe & Cie, 2005 ▶); program(s) used to solve structure: *SHELXS97* (Sheldrick, 2008 ▶); program(s) used to refine structure: *SHELXL97* (Sheldrick, 2008 ▶); molecular graphics: *SHELXTL* (Sheldrick, 2008 ▶); software used to prepare material for publication: *SHELXTL*.

## Supplementary Material

Crystal structure: contains datablocks I, global. DOI: 10.1107/S1600536809002955/cv2512sup1.cif

Structure factors: contains datablocks I. DOI: 10.1107/S1600536809002955/cv2512Isup2.hkl

Additional supplementary materials:  crystallographic information; 3D view; checkCIF report

## Figures and Tables

**Fig. 1.**
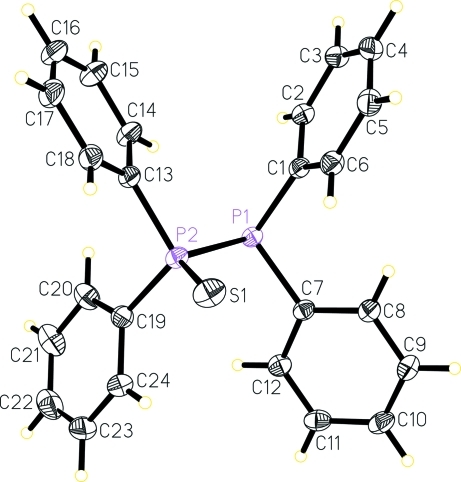
The molecular structure of the title compound showing the atom-labelling scheme and 30% probability displacement ellipsoids.

## supplementary crystallographic information

### Comment

Earlier, Gruber *et al.* (1990) reported that the only phosphorus-containing product formed from the reaction between Ph_2_PCl and thiourea was tetraphenyldiphosphane monosulfide. Bhattacharyya *et al.* (1996) reported this monosulfide formed as the by-product from the same reaction using different reaction conditions. In the present publication, we report the formation of tetraphenyldiphosphane monosulfide, which was observed as a major by-product in the reaction of Ph_2_P(S)CH_2_N(Li)Ph and ClPPh_2_. Its molecular structure (Fig. 1) shows the bond lengths and bond angles are within the normal ranges and are in accordance with the corresponding values in tetramethyldiphosphane monosulfide (Gruber *et al.*, 1990). The molecular structure of pentacarbonyl(tetraphenyldiphosphinomonosulfide-P)chromium(0) was reported earlier by Jones *et al.* (2002).

### Experimental

BuLi (0.77 ml, 1.94 mmol, 2.5 *M* in *n*-hexane) was added dropwise to a solution of Ph_2_P(S)CH_2_N(H)Ph (660 mg, 2.03 mmol) in THF (5 ml) at -78°C and this reaction mixture was stirred for 4 h while slowly warming up to -40°C. The resultant yellow solution was added to ClPPh_2_ (0.38 ml, 2.03 mmol) at 0°C in small portions *via* a cannula over a period of 20 min and followed stirring at room temperature overnight. The major part of THF was removed from the reaction mixture and over-layered with *n*-hexane to get single crystals of the title compound, which were suitable for X-ray analysis. ^31^P NMR (THF-*d*_8_): -14.11 (d, ^1^*J* = 247.3 Hz), 44.11 (d, ^1^*J* = 247.3 Hz).

### Refinement

All H atoms were placed in idealized positions (C—H = 0.95 Å) and refined using a riding model with *U*_iso_(H) fixed at 1.2 *U*_eq_(C).

### Crystal data

**Table:**

C_24_H_20_P_2_S	*F*(000) = 840
*M**_r_* = 402.40	*D*_x_ = 1.309 Mg m^−^^3^
Monoclinic, *P*2_1_/*c*	Mo *K*α radiation, λ = 0.71073 Å
Hall symbol: -P 2ybc	Cell parameters from 32981 reflections
*a* = 9.32670 (19) Å	θ = 2.0–29.6°
*b* = 13.6496 (4) Å	µ = 0.32 mm^−^^1^
*c* = 16.0484 (4) Å	*T* = 200 K
β = 91.7298 (17)°	Prism, colourless
*V* = 2042.12 (9) Å^3^	0.45 × 0.30 × 0.28 mm
*Z* = 4	

### Data collection

**Table:**

Stoe IPDS II diffractometer	5499 independent reflections
Radiation source: fine-focus sealed tube	4493 reflections with *I* > 2σ(*I*)
graphite	*R*_int_ = 0.027
rotation method scans	θ_max_ = 29.1°, θ_min_ = 2.0°
Absorption correction: numerical (*X-SHAPE*; Stoe & Cie, 2005)	*h* = −12→12
*T*_min_ = 0.892, *T*_max_ = 0.961	*k* = −18→18
38972 measured reflections	*l* = −21→21

### Refinement

**Table:**

Refinement on *F*^2^	Primary atom site location: structure-invariant direct methods
Least-squares matrix: full	Secondary atom site location: difference Fourier map
*R*[*F*^2^ > 2σ(*F*^2^)] = 0.033	Hydrogen site location: inferred from neighbouring sites
*wR*(*F*^2^) = 0.096	H-atom parameters constrained
*S* = 1.08	*w* = 1/[σ^2^(*F*_o_^2^) + (0.0564*P*)^2^ + 0.1948*P*] where *P* = (*F*_o_^2^ + 2*F*_c_^2^)/3
5499 reflections	(Δ/σ)_max_ = 0.001
244 parameters	Δρ_max_ = 0.29 e Å^−^^3^
1 restraint	Δρ_min_ = −0.28 e Å^−^^3^

### Special details

**Table:**

Geometry. All e.s.d.'s (except the e.s.d. in the dihedral angle between two l.s. planes) are estimated using the full covariance matrix. The cell e.s.d.'s are taken into account individually in the estimation of e.s.d.'s in distances, angles and torsion angles; correlations between e.s.d.'s in cell parameters are only used when they are defined by crystal symmetry. An approximate (isotropic) treatment of cell e.s.d.'s is used for estimating e.s.d.'s involving l.s. planes.
Refinement. Refinement of *F*^2^ against ALL reflections. The weighted *R*-factor *wR* and goodness of fit *S* are based on *F*^2^, conventional *R*-factors *R* are based on *F*, with *F* set to zero for negative *F*^2^. The threshold expression of *F*^2^ > σ(*F*^2^) is used only for calculating *R*-factors(gt) *etc*. and is not relevant to the choice of reflections for refinement. *R*-factors based on *F*^2^ are statistically about twice as large as those based on *F*, and *R*- factors based on ALL data will be even larger.

### Fractional atomic coordinates and isotropic or equivalent isotropic displacement parameters (Å^2^)

**Table:**

	*x*	*y*	*z*	*U*_iso_*/*U*_eq_	
C1	0.58305 (14)	0.77663 (9)	−0.01356 (8)	0.0323 (3)	
C2	0.58628 (15)	0.78402 (11)	−0.10028 (8)	0.0380 (3)	
H2A	0.5640	0.7283	−0.1337	0.046*	
C3	0.62169 (16)	0.87176 (12)	−0.13813 (10)	0.0450 (3)	
H3A	0.6232	0.8761	−0.1972	0.054*	
C4	0.65480 (16)	0.95274 (11)	−0.08978 (11)	0.0469 (3)	
H4A	0.6797	1.0128	−0.1156	0.056*	
C5	0.65188 (16)	0.94677 (11)	−0.00372 (10)	0.0439 (3)	
H5A	0.6751	1.0026	0.0293	0.053*	
C6	0.61519 (15)	0.85951 (10)	0.03445 (9)	0.0376 (3)	
H6A	0.6119	0.8561	0.0935	0.045*	
C7	0.66830 (14)	0.62119 (9)	0.10362 (7)	0.0309 (2)	
C8	0.78956 (15)	0.67684 (11)	0.12330 (9)	0.0399 (3)	
H8A	0.7999	0.7401	0.0996	0.048*	
C9	0.89570 (16)	0.64037 (12)	0.17754 (10)	0.0455 (3)	
H9A	0.9782	0.6789	0.1906	0.055*	
C10	0.88200 (16)	0.54883 (12)	0.21250 (9)	0.0436 (3)	
H10A	0.9544	0.5244	0.2499	0.052*	
C11	0.76235 (17)	0.49268 (11)	0.19287 (9)	0.0420 (3)	
H11A	0.7528	0.4294	0.2167	0.050*	
C12	0.65601 (15)	0.52821 (10)	0.13849 (8)	0.0360 (3)	
H12A	0.5745	0.4889	0.1250	0.043*	
C13	0.22346 (14)	0.75419 (10)	0.03664 (9)	0.0352 (3)	
C14	0.21864 (17)	0.73376 (12)	−0.04835 (10)	0.0461 (3)	
H14A	0.2841	0.6882	−0.0708	0.055*	
C15	0.11782 (19)	0.78017 (15)	−0.10032 (11)	0.0589 (5)	
H15A	0.1161	0.7675	−0.1586	0.071*	
C16	0.02074 (18)	0.84419 (13)	−0.06782 (10)	0.0609 (5)	
H16A	−0.0496	0.8743	−0.1034	0.073*	
C17	0.02482 (17)	0.86493 (12)	0.01602 (12)	0.0557 (4)	
H17A	−0.0422	0.9097	0.0380	0.067*	
C18	0.12649 (16)	0.82073 (11)	0.06868 (11)	0.0430 (3)	
H18A	0.1298	0.8359	0.1265	0.052*	
C19	0.28040 (15)	0.57847 (10)	0.13544 (9)	0.0379 (3)	
C20	0.21178 (18)	0.51924 (11)	0.07596 (10)	0.0459 (3)	
H20A	0.1989	0.5421	0.0203	0.055*	
C21	0.1620 (2)	0.42703 (13)	0.09741 (13)	0.0589 (5)	
H21A	0.1140	0.3873	0.0568	0.071*	
C22	0.1824 (2)	0.39341 (13)	0.17769 (15)	0.0642 (5)	
H22A	0.1500	0.3299	0.1922	0.077*	
C23	0.2495 (2)	0.45130 (16)	0.23683 (14)	0.0675 (6)	
H23A	0.2627	0.4275	0.2922	0.081*	
C24	0.29853 (18)	0.54423 (14)	0.21697 (11)	0.0532 (4)	
H24A	0.3439	0.5841	0.2585	0.064*	
P1	0.53134 (3)	0.65612 (2)	0.024981 (19)	0.03019 (9)	
P2	0.35427 (4)	0.69731 (2)	0.10811 (2)	0.03240 (9)	
S1	0.39983 (5)	0.77769 (3)	0.20569 (2)	0.04904 (11)	

### Atomic displacement parameters (Å^2^)

**Table:**

	*U*^11^	*U*^22^	*U*^33^	*U*^12^	*U*^13^	*U*^23^
C1	0.0296 (6)	0.0325 (6)	0.0344 (6)	0.0026 (5)	−0.0032 (5)	0.0022 (5)
C2	0.0367 (7)	0.0421 (7)	0.0350 (6)	0.0007 (6)	−0.0036 (5)	0.0036 (5)
C3	0.0396 (8)	0.0531 (8)	0.0423 (7)	0.0007 (6)	0.0002 (6)	0.0132 (6)
C4	0.0374 (7)	0.0414 (8)	0.0619 (9)	0.0012 (6)	0.0019 (6)	0.0156 (7)
C5	0.0371 (7)	0.0336 (7)	0.0608 (9)	0.0017 (6)	−0.0018 (6)	−0.0012 (6)
C6	0.0363 (7)	0.0359 (7)	0.0404 (7)	0.0015 (5)	−0.0020 (5)	−0.0015 (5)
C7	0.0305 (6)	0.0328 (6)	0.0294 (5)	0.0062 (5)	−0.0001 (4)	−0.0012 (4)
C8	0.0347 (7)	0.0404 (7)	0.0441 (7)	−0.0002 (5)	−0.0050 (6)	0.0058 (6)
C9	0.0317 (7)	0.0548 (9)	0.0494 (8)	0.0009 (6)	−0.0065 (6)	0.0038 (7)
C10	0.0391 (7)	0.0533 (8)	0.0383 (7)	0.0160 (6)	−0.0034 (6)	0.0020 (6)
C11	0.0511 (8)	0.0361 (7)	0.0390 (7)	0.0121 (6)	0.0018 (6)	0.0043 (5)
C12	0.0392 (7)	0.0312 (6)	0.0373 (6)	0.0045 (5)	−0.0005 (5)	−0.0015 (5)
C13	0.0304 (6)	0.0321 (6)	0.0430 (7)	0.0011 (5)	−0.0030 (5)	0.0059 (5)
C14	0.0389 (7)	0.0547 (9)	0.0438 (7)	0.0065 (7)	−0.0107 (6)	0.0024 (6)
C15	0.0479 (9)	0.0735 (12)	0.0542 (9)	0.0031 (8)	−0.0182 (8)	0.0125 (8)
C16	0.0405 (8)	0.0593 (10)	0.0816 (13)	0.0032 (8)	−0.0181 (8)	0.0254 (9)
C17	0.0359 (8)	0.0426 (8)	0.0887 (13)	0.0083 (6)	0.0001 (8)	0.0139 (8)
C18	0.0343 (7)	0.0352 (7)	0.0595 (9)	0.0035 (5)	0.0032 (6)	0.0068 (6)
C19	0.0321 (6)	0.0387 (7)	0.0432 (7)	0.0083 (5)	0.0062 (5)	0.0076 (5)
C20	0.0526 (9)	0.0380 (7)	0.0481 (8)	0.0001 (6)	0.0162 (7)	−0.0033 (6)
C21	0.0642 (11)	0.0407 (8)	0.0733 (11)	−0.0032 (8)	0.0278 (9)	−0.0076 (8)
C22	0.0558 (11)	0.0419 (9)	0.0963 (15)	0.0086 (8)	0.0248 (10)	0.0192 (9)
C23	0.0510 (10)	0.0739 (13)	0.0776 (12)	0.0079 (9)	0.0020 (9)	0.0428 (11)
C24	0.0423 (8)	0.0632 (10)	0.0538 (9)	0.0029 (7)	−0.0049 (7)	0.0224 (8)
P1	0.03128 (16)	0.03040 (16)	0.02864 (15)	0.00166 (12)	−0.00294 (12)	−0.00140 (11)
P2	0.03193 (17)	0.03396 (17)	0.03117 (16)	0.00544 (13)	−0.00136 (12)	0.00023 (12)
S1	0.0586 (2)	0.0524 (2)	0.03591 (18)	0.00869 (18)	−0.00217 (16)	−0.01034 (15)

### Geometric parameters (Å, °)

**Table:**

C1—C6	1.3962 (18)	C13—C14	1.392 (2)
C1—C2	1.3966 (18)	C13—P2	1.8228 (13)
C1—P1	1.8273 (13)	C14—C15	1.391 (2)
C2—C3	1.387 (2)	C14—H14A	0.9500
C2—H2A	0.9500	C15—C16	1.3727 (19)
C3—C4	1.380 (2)	C15—H15A	0.9500
C3—H3A	0.9500	C16—C17	1.3743 (19)
C4—C5	1.385 (2)	C16—H16A	0.9500
C4—H4A	0.9500	C17—C18	1.389 (2)
C5—C6	1.387 (2)	C17—H17A	0.9500
C5—H5A	0.9500	C18—H18A	0.9500
C6—H6A	0.9500	C19—C20	1.392 (2)
C7—C8	1.3907 (19)	C19—C24	1.395 (2)
C7—C12	1.3931 (18)	C19—P2	1.8210 (15)
C7—P1	1.8316 (12)	C20—C21	1.388 (2)
C8—C9	1.3905 (19)	C20—H20A	0.9500
C8—H8A	0.9500	C21—C22	1.375 (3)
C9—C10	1.377 (2)	C21—H21A	0.9500
C9—H9A	0.9500	C22—C23	1.372 (3)
C10—C11	1.382 (2)	C22—H22A	0.9500
C10—H10A	0.9500	C23—C24	1.389 (3)
C11—C12	1.3887 (19)	C23—H23A	0.9500
C11—H11A	0.9500	C24—H24A	0.9500
C12—H12A	0.9500	P1—P2	2.2263 (5)
C13—C18	1.391 (2)	P2—S1	1.9486 (5)
			
C6—C1—C2	118.73 (13)	C13—C14—H14A	120.1
C6—C1—P1	126.67 (10)	C16—C15—C14	120.26 (16)
C2—C1—P1	114.59 (10)	C16—C15—H15A	119.9
C3—C2—C1	120.75 (14)	C14—C15—H15A	119.9
C3—C2—H2A	119.6	C15—C16—C17	120.27 (15)
C1—C2—H2A	119.6	C15—C16—H16A	119.9
C4—C3—C2	119.83 (14)	C17—C16—H16A	119.9
C4—C3—H3A	120.1	C16—C17—C18	120.30 (15)
C2—C3—H3A	120.1	C16—C17—H17A	119.9
C3—C4—C5	120.19 (14)	C18—C17—H17A	119.9
C3—C4—H4A	119.9	C17—C18—C13	119.88 (16)
C5—C4—H4A	119.9	C17—C18—H18A	120.1
C4—C5—C6	120.24 (14)	C13—C18—H18A	120.1
C4—C5—H5A	119.9	C20—C19—C24	119.26 (14)
C6—C5—H5A	119.9	C20—C19—P2	121.44 (11)
C5—C6—C1	120.26 (13)	C24—C19—P2	119.23 (12)
C5—C6—H6A	119.9	C21—C20—C19	120.44 (16)
C1—C6—H6A	119.9	C21—C20—H20A	119.8
C8—C7—C12	118.85 (12)	C19—C20—H20A	119.8
C8—C7—P1	124.04 (10)	C22—C21—C20	119.80 (19)
C12—C7—P1	116.73 (10)	C22—C21—H21A	120.1
C9—C8—C7	120.35 (14)	C20—C21—H21A	120.1
C9—C8—H8A	119.8	C23—C22—C21	120.22 (17)
C7—C8—H8A	119.8	C23—C22—H22A	119.9
C10—C9—C8	120.45 (14)	C21—C22—H22A	119.9
C10—C9—H9A	119.8	C22—C23—C24	120.92 (17)
C8—C9—H9A	119.8	C22—C23—H23A	119.5
C9—C10—C11	119.63 (13)	C24—C23—H23A	119.5
C9—C10—H10A	120.2	C23—C24—C19	119.34 (18)
C11—C10—H10A	120.2	C23—C24—H24A	120.3
C10—C11—C12	120.40 (13)	C19—C24—H24A	120.3
C10—C11—H11A	119.8	C1—P1—C7	106.37 (6)
C12—C11—H11A	119.8	C1—P1—P2	100.58 (5)
C11—C12—C7	120.31 (13)	C7—P1—P2	99.63 (4)
C11—C12—H12A	119.8	C19—P2—C13	106.26 (6)
C7—C12—H12A	119.8	C19—P2—S1	112.51 (5)
C18—C13—C14	119.41 (13)	C13—P2—S1	113.05 (5)
C18—C13—P2	118.41 (11)	C19—P2—P1	102.26 (5)
C14—C13—P2	122.18 (11)	C13—P2—P1	103.00 (5)
C15—C14—C13	119.85 (16)	S1—P2—P1	118.43 (2)
C15—C14—H14A	120.1		
			
C6—C1—C2—C3	0.4 (2)	C22—C23—C24—C19	−0.7 (3)
P1—C1—C2—C3	179.04 (11)	C20—C19—C24—C23	0.9 (2)
C1—C2—C3—C4	0.3 (2)	P2—C19—C24—C23	−176.34 (14)
C2—C3—C4—C5	−0.4 (2)	C6—C1—P1—C7	−52.24 (13)
C3—C4—C5—C6	−0.2 (2)	C2—C1—P1—C7	129.30 (10)
C4—C5—C6—C1	1.0 (2)	C6—C1—P1—P2	51.19 (12)
C2—C1—C6—C5	−1.1 (2)	C2—C1—P1—P2	−127.26 (10)
P1—C1—C6—C5	−179.48 (11)	C8—C7—P1—C1	−3.34 (14)
C12—C7—C8—C9	−0.6 (2)	C12—C7—P1—C1	−176.18 (10)
P1—C7—C8—C9	−173.34 (12)	C8—C7—P1—P2	−107.46 (12)
C7—C8—C9—C10	−0.1 (2)	C12—C7—P1—P2	79.70 (10)
C8—C9—C10—C11	0.5 (2)	C20—C19—P2—C13	41.29 (14)
C9—C10—C11—C12	−0.3 (2)	C24—C19—P2—C13	−141.56 (12)
C10—C11—C12—C7	−0.5 (2)	C20—C19—P2—S1	165.51 (11)
C8—C7—C12—C11	0.9 (2)	C24—C19—P2—S1	−17.34 (14)
P1—C7—C12—C11	174.15 (11)	C20—C19—P2—P1	−66.36 (12)
C18—C13—C14—C15	0.3 (2)	C24—C19—P2—P1	110.79 (12)
P2—C13—C14—C15	−179.17 (13)	C18—C13—P2—C19	97.09 (12)
C13—C14—C15—C16	−1.6 (3)	C14—C13—P2—C19	−83.43 (14)
C14—C15—C16—C17	1.7 (3)	C18—C13—P2—S1	−26.79 (13)
C15—C16—C17—C18	−0.5 (3)	C14—C13—P2—S1	152.68 (11)
C16—C17—C18—C13	−0.8 (2)	C18—C13—P2—P1	−155.79 (10)
C14—C13—C18—C17	0.9 (2)	C14—C13—P2—P1	23.68 (13)
P2—C13—C18—C17	−179.60 (12)	C1—P1—P2—C19	171.48 (6)
C24—C19—C20—C21	−0.1 (2)	C7—P1—P2—C19	−79.70 (6)
P2—C19—C20—C21	177.07 (13)	C1—P1—P2—C13	61.35 (6)
C19—C20—C21—C22	−0.9 (3)	C7—P1—P2—C13	170.16 (6)
C20—C21—C22—C23	1.1 (3)	C1—P1—P2—S1	−64.24 (5)
C21—C22—C23—C24	−0.3 (3)	C7—P1—P2—S1	44.58 (5)
